# Exosomes from Plasma of Neuroblastoma Patients Contain Doublestranded DNA Reflecting the Mutational Status of Parental Tumor Cells

**DOI:** 10.3390/ijms22073667

**Published:** 2021-04-01

**Authors:** Chiara Degli Esposti, Barbara Iadarola, Simone Maestri, Cristina Beltrami, Denise Lavezzari, Martina Morini, Patrizia De Marco, Giovanni Erminio, Alberto Garaventa, Federico Zara, Massimo Delledonne, Marzia Ognibene, Annalisa Pezzolo

**Affiliations:** 1 Dipartimento di Biotecnologie, Università degli Studi di Verona, 37134 Verona, Italy; chiara.degliesposti@univr.it (C.D.E.); barbara.iadarola@univr.it (B.I.); simone.maestri@univr.it (S.M.); cristina.beltrami_01@univr.it (C.B.); denise.lavezzari@univr.it (D.L.); massimo.delledonne@univr.it (M.D.); 2 Laboratorio di Biologia Molecolare, IRCCS Giannina Gaslini, 16147 Genova, Italy; martinamorini@gaslini.org; 3 U.O.C. Genetica Medica, IRCCS Giannina Gaslini, 16147 Genova, Italy; patriziademarco@gaslini.org (P.D.M.); federicozara@gaslini.org (F.Z.); 4 Epidemiologia e Biostatistica, IRCCS Giannina Gaslini, 16147 Genova, Italy; giovannierminio@gaslini.org; 5 Divisione di Oncologia, IRCCS Giannina Gaslini, 16147 Genova, Italy; albertogaraventa@gaslini.org; 6 IRCCS Giannina Gaslini, 16147 Genova, Italy; annalisapezzolo@gaslini.org

**Keywords:** neuroblastoma, exosomes, exo-DNA, *ALK*, genotypability, tumor mutation load

## Abstract

Neuroblastoma (NB) is an aggressive infancy tumor, leading cause of death among preschool age diseases. Here we focused on characterization of exosomal DNA (exo-DNA) isolated from plasma cell-derived exosomes of neuroblastoma patients, and its potential use for detection of somatic mutations present in the parental tumor cells. Exosomes are small extracellular membrane vesicles secreted by most cells, playing an important role in intercellular communications. Using an enzymatic method, we provided evidence for the presence of double-stranded DNA in the NB exosomes. Moreover, by whole exome sequencing, we demonstrated that NB exo-DNA represents the entire exome and that it carries tumor-specific genetic mutations, including those occurring on known oncogenes and tumor suppressor genes in neuroblastoma (*ALK*, *CHD5*, *SHANK2*, *PHOX2B*, *TERT*, *FGFR1*, and *BRAF*). NB exo-DNA can be useful to identify variants responsible for acquired resistance, such as mutations of *ALK*, *TP53*, and *RAS*/*MAPK* genes that appear in relapsed patients. The possibility to isolate and to enrich NB derived exosomes from plasma using surface markers, and the quick and easy extraction of exo-DNA, gives this methodology a translational potential in the clinic. Exo-DNA can be an attractive non-invasive biomarker for NB molecular diagnostic, especially when tissue biopsy cannot be easily available.

## 1. Introduction

Neuroblastoma (NB), the most common extracranial solid tumor occurring in childhood, arises from neural crest cells of the sympathetic nervous system [1,2]. NB presents a high clinical heterogeneity that ranges from spontaneous regression to aggressive metastasis [3,4]. NB patients diagnosed with metastatic disease, or older than 18 months, or when the tumor carries genomic amplification of *MYCN* oncogene and/or segmental chromosome abnormalities, are considered to be at high risk of death [5]. The other NB patients are classified as intermediate or low risk. Besides to *MYCN* amplification, the rearrangements of *TERT* gene, encoding the catalytic subunit of telomerase, or inactivating mutations in *ATRX* and *ARID1A* loci, encoding chromatin-remodeling proteins, have been detected mainly in high-risk NB [6,7,8,9,10]. Moreover, NB shows recurrent mutations in *ALK* gene that encodes a receptor tyrosine kinase [11,12]. Mutations in genes of the *RAS* and *p53* pathways have been described in relapsed NB, and they occur in both high- and non-high-risk tumors although at lower frequencies in the latter group [13,14]. Other somatic mutations have been detected in primary NB albeit rarely (<5%), including nucleotide alterations in *PTPRD* gene encoding a protein tyrosine phosphatase, in *ODZ3* gene encoding a transmembrane protein involved in neuronal development, and in *PTPN11* gene encoding a receptor tyrosine kinase [8,9].

Recently, liquid biopsies have been mostly employed in the detection of tumoral biomarkers allowing a non-invasive diagnosis especially in case the tissue biopsy was difficult to collect [15]. Two of the most important biomarkers classes in the field of liquid biopsy are the cell-free DNA (cfDNA) and exosomes [16]. Exosomes are small extracellular membrane vesicles of endocytic origin, secreted by most cells and playing a central role in intercellular communications [17,18]. Exosomes are released by NB cells too [19,20], and it has been demonstrated that exosomal DNA (exo-DNA) derived from tumor cells showed 20-fold more DNA than the one isolated from fibroblasts [21]. As reported by Khalert et al., exosomes contain double stranded (ds) DNA that could be >10 Kb long [22]. On the contrary, cfDNA is generally more fragmented (between 120–220 bp), maybe due to its nucleosomal association [23]. Furthermore, the tumor-derived fraction of cfDNA, known as circulating tumor DNA (ctDNA), is usually even more fragmented than healthy cfDNA, with a size between 90 and 150 bp [24].

In this study, given the recent findings on the biological content of exosomes, including chromosomal DNA [21,25], we focused on characterization of the NB exo-DNA and its potential use as a non-invasive biomarker for screening of somatic mutations present in the parental cells.

## 2. Results

### 2.1. Patients Characteristics

Nineteen NB patients were enrolled as described in Section 4. Primary tumor DNA from fifteen patients for which there was enough available material, was firstly analyzed by array-CGH, and disclosed a genomic profile with multiple segmental chromosome gains or losses in twelve specimens, or with only numerical chromosome aberrations in three samples (Table 1).

### 2.2. Exosomes from NB Patients Contain Genomic DsDNA Covering All Chromosomes

Exosomes were purified from peripheral blood samples of the nineteen NB patients at onset of the disease and, in four cases, at the time of relapse too. The NB cell-derived exosomes are nanoparticles already well characterized in terms of size (30–150 nm) [26], and positivity for the GD2 marker, specifically expressed on NB cells surface, as we described in our previous manuscript [19]. In that work, we analyzed NB plasma-derived exosomes by flow cytometry using the anti-tetraspanin CD9 mAb, recognizing the typical exosomal surface marker, and the anti-GD2 mAb to confirm their purity. This analysis showed that about 85–90% of NB-derived exosomes expressed CD9, and that the mean percentage of GD2^+^ exosomes was over 50%. Therefore, we demonstrated that plasma samples from NB patients contain NB cell-derived exosomes [19]. Exosomes were treated with DNase I, before extracting the exo-DNA, to eliminate any external DNA contamination.

**Figure 1:**
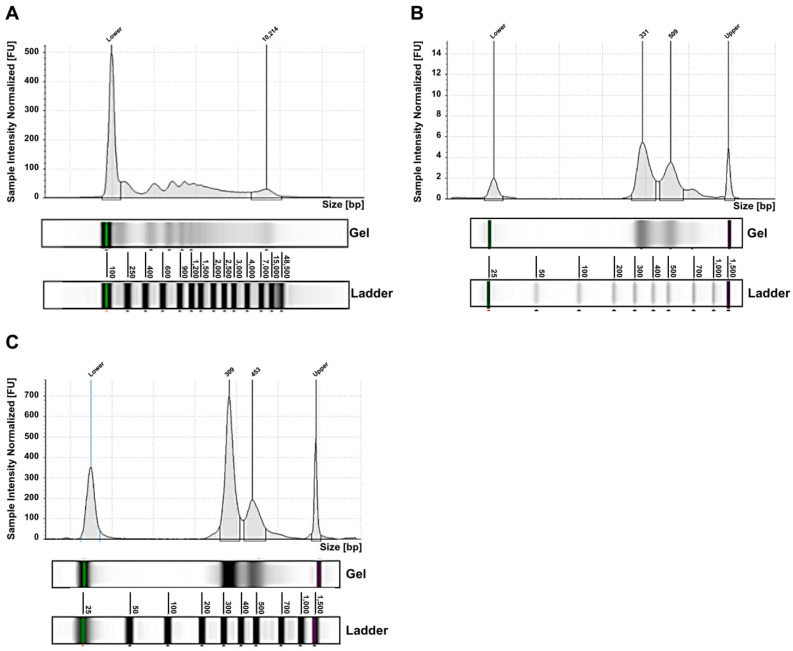
Exo-DNA size profiling (Tape Station 4150). (**A**) Representative electropherogram of exo-DNA. (**B**) Exo-DNA library. (**C**) Target enrichment capture of exo-DNA (FU = fluorescence unit; bp = base pair).

For the NB cases analyzed by whole-exome sequencing (WES), in addition to exo-DNA, the other available DNA specimens were the tumor DNA extracted from needle aspiration or micro-biopsy tissue (for ten patients only) and the genomic blood DNA (gDNA). Fluorimetric sizing analysis of exo-DNA showed a size distribution ranging from 186 bp to 48 kb, with distinct peaks (Figure 1A). Exo-DNA was mechanically fragmented in order to obtain the desired insert size (280 bp) however, due to its initial profile, the final library profiles exhibited two characteristic peaks, the first one of 310–340 bp and the second one of 450–500 bp (Figure 1B). After exome enrichment capture, this size distribution was maintained (Figure 1C).

**Figure 2:**
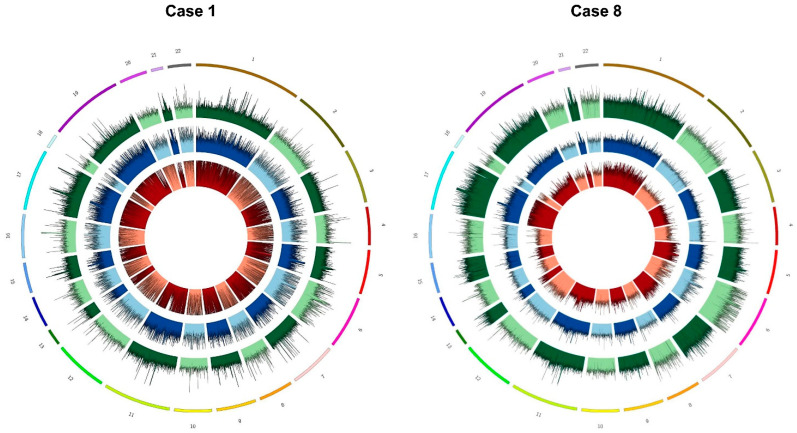
Circular view of chromosomes coverage for two representative cases. Each color represents a different DNA specimen: Tumor DNA (red), exo-DNA at onset (blue), exo-DNA at relapse (green).

Exome enriched libraries performed for all nineteen NB cases were sequenced on an Illumina Novaseq 6000, using the 150 bp paired-end mode. Considering the fragments obtained from sequencing and their alignment on the reference genome GRCh38/hg38, the median sequenced fragment size of exo-DNA specimens reached 191 bp, confirming a lower fragmentation pattern typically observed for cfDNA. Tumor biopsy and genomic DNA specimens resulted in a fragment length of 306 and 308 bp, respectively, in line with the expected sequenced size of WES libraries. Observed coverage for all exo-DNA specimens was greater than 50×, with a median value of 110×; median coverage observed for gDNA and tumor DNA was of 147× and 131×, respectively. More specifically, the percentage of the target covered by at least 5 (%5×: 97.99%) and 10 reads (%10×: 97.79%) in exo-DNA reflected similar performance of WES on gDNA and tumor samples (Supplementary Table S1). Indeed, the coverage was uniform along all chromosomes of exo-DNA specimens (Figure 2).

Most importantly, it was possible to genotype the whole exome through the analysis of exo-DNA (the median genotypability value (%PASS) obtained on the entire target region (design) was 97.8) (Supplementary Table S1). In particular, the integration of unique molecular identifiers (UMIs) in the sample preparation, as described in Section 4, allowed a more accurate identification of PCR duplicated fragments. Moreover, the removal of fragments deriving from repetitive regions of the genome using the UMI’s bioinformatics pipeline increased the genotypability along all the target regions. These results showed higher genotypability of the exome (more than 1%) respect to previously reported WES analyses on genomic DNA, with a sequenced fragment length of 350 bp [27]. To be noticed, the exosomes genotype was higher (more than 2%) than in exomes, with even shorter fragment sizes (200 bp) and read length (75 bp) [27]. Given the large size of the entire target region, the genotypability for each chromosome in the different DNA specimens was investigated, looking for genotypability differences. No substantial differences emerged among chromosomes within the same DNA specimen, confirming a high genotypability distributed along the entire exome. The median of the samples for each tumor DNA, exo-DNA at onset, exo-DNA at relapse is reported in Table 2.

By comparing the genotype data between exo-DNA and gDNA, we noted that the genotypability of exo-DNA (in particular at onset) was equal if not higher than control. The coding regions of autosomal chromosomes were all well represented in the exo-DNA at onset (%PASS > 73% with a mean of 90.86%), as was for gDNA (%PASS > 71% with a mean of 87.24%). The exo-DNA at relapse, which in some cases had lower DNA amount, showed a %PASS above 70% for all chromosomes with an average of 86.4%. Tumor DNA had a good %PASS (>72%) too, with an average of 89.46%.

### 2.3. Tumor DNA Content in Exo-DNA Samples

**Figure 3:**
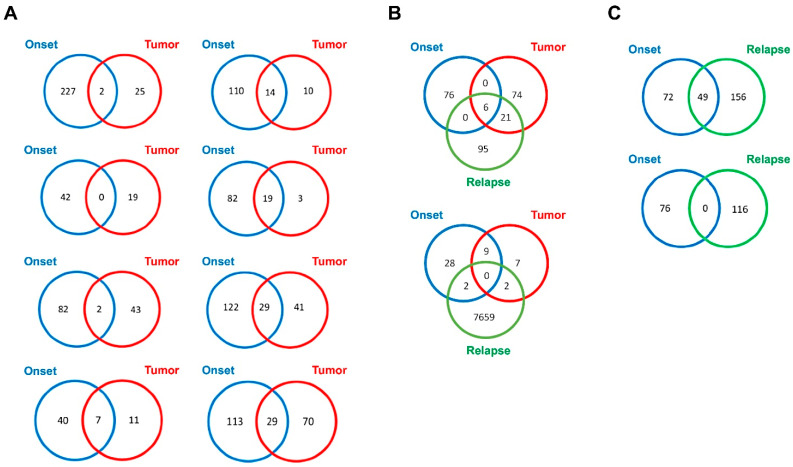
Venn diagrams of somatic single nucleotide variants (SNVs) shared by exo-DNA and tumor DNA. (**A**) SNVs in common between exo-DNA at onset and the corresponding tumor DNA. (**B**) SNVs in common among exo-DNA at onset, exo-DNA at relapse, and the corresponding tumor DNA. (**C**) SNVs in common between exo-DNA at onset and exo-DNA at relapse.

The somatic single nucleotide variants (SNVs) identified in the tumor DNA, exo-DNA at onset and at relapse were then evaluated. For this purpose, the number of SNVs in common between the different DNA specimens available for each case was calculated. In ten NB cases with available exo-DNA at onset and the corresponding tumor DNA, a higher overall number of SNVs was identified in the exo-DNA compared to tumor DNA (Figure 3A,B). This could mean that SNVs detected in exo-DNA arose from tumor sites different from the tumor DNA site of extraction, highlighting a spatial genetic heterogeneity in NB. In the NB cases with all DNA specimens available (exo-DNA at onset, exo-DNA at relapse, tumor DNA) many SNVs were found to be exclusive of a single specimen, with low concordance among them (Figure 3B). The number of SNVs in common between exo-DNA at onset and exo-DNA at relapse remained low, with a greater number of variants identified for the relapse (Figure 3C).

**Figure 4:**
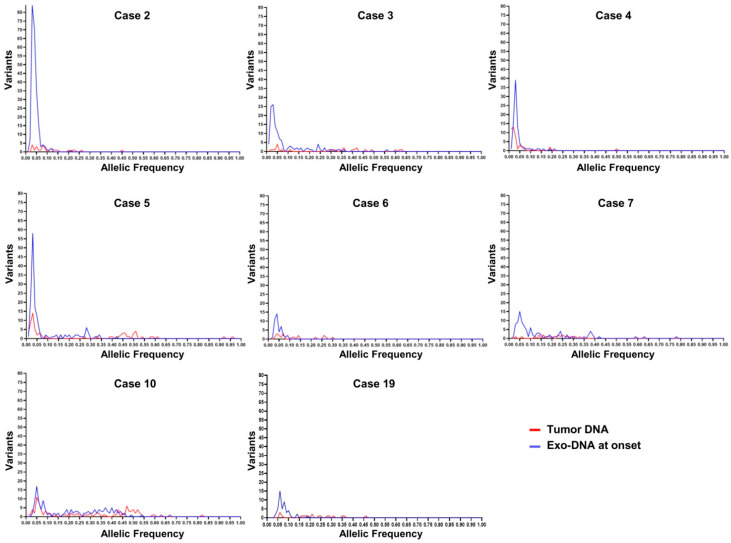
Somatic SNVs frequency in neuroblastoma (NB) cases with exo-DNA at onset and tumor DNA.

**Figure 5:**
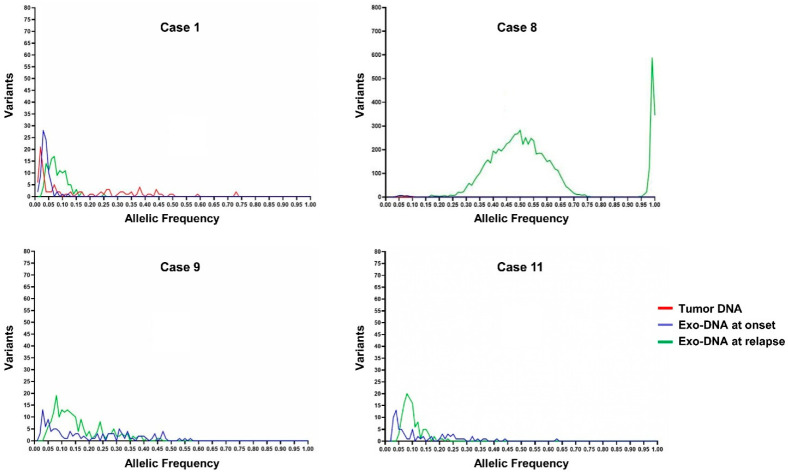
Somatic SNVs frequency in NB cases with exo-DNA at relapse.

Subsequently, the frequency of the SNVs identified in the different DNA specimens was investigated. By observing the frequency graphs of the variants identified in the NB cases with available exo-DNA at onset and the corresponding tumor DNA, we identified the presence of clones with lower frequency SNVs in the exo-DNA compared to the tumor DNA (Figure 4). The exo-DNA at relapse had SNVs with higher frequency than the exo-DNA at onset, probably as indicator of clonal evolution (Figure 5). NB cases with only exo-DNA at onset available, showed the same trend seen for other cases, with most of the identified SNVs having low frequency (Supplementary Figure S1).

**Figure 6:**
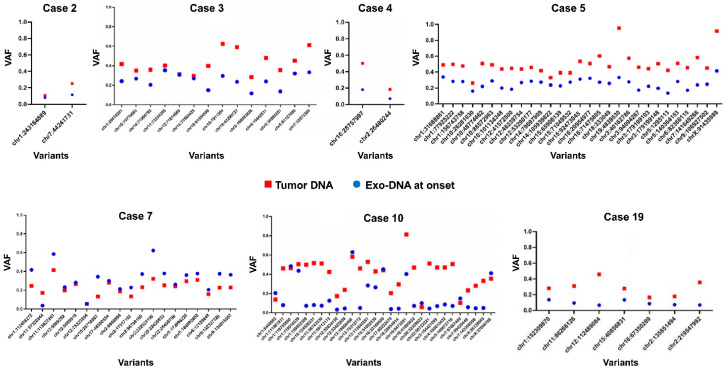
Frequency of the somatic SNVs in common between tumor DNA and exo-DNA at onset (VAF = variant allele frequency).

To investigate if the dsDNA extracted from NB cell-derived exosomes reflected the mutational status of their parental tumor cells, we performed further analysis on the somatic SNVs found in common between different DNA specimens of the same case, evaluating their allelic frequency. Considering the NB cases with available tumor DNA and exo-DNA at onset, we observed that SNVs frequency in the exo-DNA at onset was generally lower than in tumor DNA, probably indicating less tumor content in exo-DNA (Figure 6).

**Figure 7:**
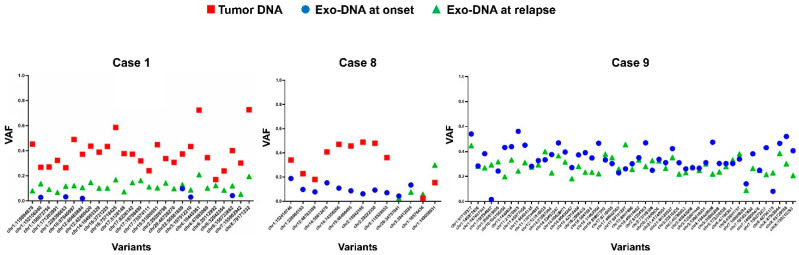
Frequency of the somatic SNVs in common between exo-DNA at relapse and tumor DNA/exo-DNA at onset. (VAF = variant allele frequency).

In the NB cases with all DNA specimens available, tumor SNVs appeared to have a higher frequency than both exo-DNA at onset and exo-DNA at relapse, as expected (Figure 7). The last two SNVs in case 8 displayed an abnormal behavior, with a frequency in the tumor DNA lower than in exo-DNA at relapse. The exo-DNA at relapse often showed higher frequency SNVs than the exo-DNA at onset, probably indicating some sort of clonal evolution inducing the relapse itself and the consequent poor prognosis of the patient. The WES results therefore demonstrated that parental tumor SNVs were detectable in the exo-DNA even if with lower frequency.

### 2.4. Exo-DNA-Derived Tumor Mutation Load

The tumor mutation load (TML) resulting from the non-synonymous variants number per megabase (Mb) identified in the target region [28], was considered for the exo-DNA and the tumor DNA. TML value was calculated by WES data for each available specimen, then deriving the median for each type of specimen. Median TML was 1.5 non-synonymous variants per Mb in exo-DNA at onset, 3.55 non-synonymous variants per Mb in exo-DNA at relapse and 0.45 non-synonymous variants per Mb in tumor DNA. These results indicated that tumor DNA TML was in line with the known value already reported for NB, that is a median frequency of 0.48 non-synonymous variants per Mb [9]. Interestingly, TML of exo-DNA both at onset and at relapse was higher than in tumor DNA. The higher TML value observed in the exosomes at onset is probably due to the exo-DNA somatic SNVs not detectable in tumor DNA. Our cohort of NB patients with high TML value in exo-DNA had a worse outcome in comparison with patients with lower values.

### 2.5. Exo-DNA Contains SNVs of Known Neuroblastoma Driver Genes

The annotation of the SNVs with tumor databases had allowed the classification of many of them from a clinical point of view [29,30]. Main analysis identified somatic SNVs associated with NB in many of the exo-DNAs in the COSMIC somatic mutations database. Known driver and tumor suppressor genes in NB were frequently mutated in the exo-DNA (*ALK*, *CHD5*, *SHANK2*, *PHOX2B*, *TERT*, *FGFR1*, and *BRAF*). In Table 3, we reported the somatic SNVs identified in the different DNA specimens, for each analyzed NB case. To notice, some clinical variants of *ALK* gene were present in several cases.

**Figure 8:**
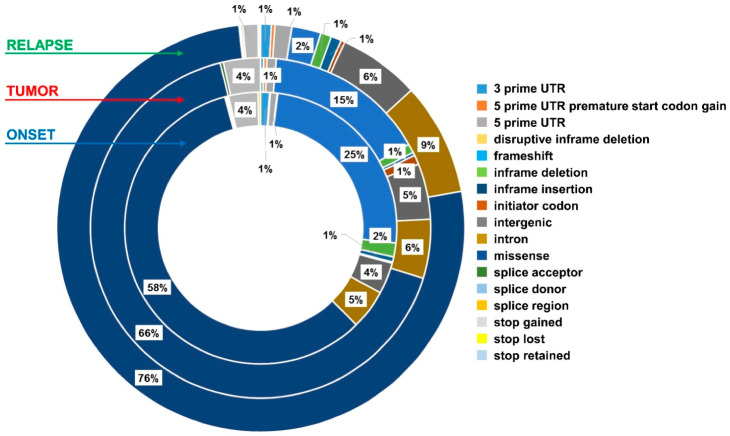
Mutation types in exo-DNA and in tumor DNA.

The majority of the identified somatic SNVs are missense variants, confirming what has already been reported in the literature (Figure 8).

### 2.6. Most Frequently Mutated Genes in Exo-DNA and in Tumor DNA

Next, we evaluated whether in this NB patient cohort there were frequently mutated genes different between exo-DNA and tumor DNA samples. Table 4 reports, for each DNA specimen, the list of the 20 most significantly enriched genes. The results showed that most mutated genes did not always match in the different DNA specimens. In particular, the exo-DNA at onset displayed a more significant enrichment than the exo-DNA at relapse and the tumor DNA. In particular, *TYW1* gene was the most mutated gene in exo-DNA at onset, while *AHNAK2* and *ALK* genes were the most mutated genes in the exo-DNA at relapse and in primary tumor DNA, respectively. *ALK* is one of the most significantly mutated gene in the exo-DNA at onset and in tumor DNA. Particularly mutated regions (hotspots) have also been identified in exo-DNA at onset within the listed genes (Table 5), and chromosome 7 appeared to be the most mutated one. Among these genes, we found new ones, not already associated with NB, that were frequently mutated in this cohort of patients, such as *TYW1* and *DPP6.*

### 2.7. Identified Copy Number Variations Involved in NB Oncogenesis

All copy number variations (CNVs) found within the known driver genes related to NB or to other tumors, were investigated [4,8,9]. We found that some genes of interest displayed CNVs in different DNA specimens (Table 6; Supplementary Table S2). In particular, CNVs affected genes involved in the RAS pathway (*PTPN11*, *NF1*, *KRAS*, and *BRAF*) and other genes already detected in SNVs analysis (*ALK*, and *SHANK2*). For the cases with multiple available DNA specimens, it was not always possible to identify tumor CNVs in the exo-DNA, anyway a few CNVs have been detected in multiple DNA specimens. For example, *MYCN* amplification has been observed in tumor DNA and in exo-DNA both at onset and at relapse. For patients with only exo-DNA at onset available, many known NB driver genes were duplicated or deleted. Notably, *MYCN*, *TERT*, and *SHANK2* genes showed in some cases a very high copy number (greater than 10).

## 3. Discussion

Exosomes are extracellular membrane vesicles with a size ranging from 30 to 150 nm. These vesicles are released by most cells, including tumor cells, and therefore can be used as a source of DNA, messenger RNA, micro-RNA and proteins [7,18,21,25]. Circulating exosomes represent new components of the tumor microenvironment, and constitute a reliable source of biomarkers that allows real-time monitoring of the disease [19,20,26]. Exosomes purified from peripheral blood sample only require a simple blood draw, which can be repeated multiple times with minimal impact for patients. The use of exosomes as source of non-invasive biomarkers makes possible to test tumor sites more frequently. The advancement of personalized medicine in oncology is based on the development of non-invasive approaches for stratification, diagnosis, monitoring of therapeutic response, and detection of patients’ minimal residual disease. Exosomes represent this type of experimental approach, and are able to provide reliable and solid information on disease, metabolism and on the genomic profile of parental tumor cells.

Therapeutic stratification of NB patients is based on clinical and molecular risk factors that correlate with prognosis, and it is essential for treatment [1,2,3,4,5]. However, patients with similar clinical-pathological parameters who receive the same treatment may have different clinical courses. For this reason, it is important to identify new and better prognostic markers capable of directing therapy towards personalized medicine. Since approximately 50% of high-risk NB patients die despite treatment, new strategies are needed to improve therapy. The clinical, radiological and biological markers currently used in clinical routine are not able to predict in an early and accurate way a possible therapeutic failure that would justify a change in treatment. Exosomes represent an interesting method to obviate the limited amount of tumor tissue obtainable by needle aspiration or micro-biopsy, as well as an alternative for patients who cannot undergo tissue biopsy.

Our study provides evidence that exosomes isolated from plasma samples of NB patients contain double stranded tumor DNA spanning all chromosomes. We suggest a new source of circulating DNA in addition to plasma derived cell-free DNA (cfDNA) resulting from cell lysis or apoptosis [15,31,32]. Indeed, the substantial difference between exo-DNA and cfDNA is that the former derives from living cells, whereas the latter comes, in large part, from dead ones. Therefore, the exo-DNA can better reflect the malignant and aggressive tumor cells populations, and better highlights on tumor dynamics. Potentially, the exo-DNA is much more clinically useful than the cfDNA, which is diluted in plasma or in other body fluids. The exo-DNA is stable and protected inside vesicles, that also carry tumor related miRNA [19], and proteins [33], which have been used successfully as tumor biomarkers. Moreover, the exosomes can be isolated and enriched by exploiting their specific surface markers, thus enriching in exo-DNA too.

We demonstrated by WES that exo-DNA covers the whole exome, obtaining results very similar to those previously reported by WES on genomic DNA [27]. More specifically, the fragmentation pattern obtained for the exosomes could allow sequencing longer reads (150 bp), compared to cfDNA, that appears more fragmented. This, in combination with the use of UMIs during sample preparation, leads to an accurate analysis of the entire exome starting from exo-DNA sequencing. Noteworthy, the NB exo-DNA displays mutations identical to the parental tumor cells DNA, despite the limited number of analyzed samples. Some low frequency variants (e.g., 2/3 reads to support the variant), identified in one specimen only, were present in another specimen of the same case at a lower frequency (e.g., 1 read to support the variant). While in the first specimen the variant was called thanks to the higher number of supporting reads, in the other specimens the variant was not identified. This could have led to the discovery of fewer variants in common between different DNA specimens, under-estimating the tumoral variants present in the exo-DNA. Differences existing between tumoral and exosome specimens’ coverage may have influenced the variant calling. The exo-DNA contains information regarding somatic SNVs and CNVs of *ALK*, *MYCN*, *PTPN11*, *ATRX*, *SMARCA4*, *TP53*, *NF1*, *CHD5*, *TERT*, *BRAF*, *SHANK2*, *KRAS*, and *CDKN1B* genes present in their parental cells too. The frequency of the somatic SNVs in the exo-DNA at onset is generally lower than in tumor DNA, probably indicating clonal mutational events in the exo-DNA. We identified SNVs in the exo-DNA that were not present in tumor DNA at onset of disease, in particular mutations of *ALK*, *ATRX*, *NF1*, and *TERT* genes, probably coming from other tumor sites. The exosomes derive from cells of distinct tumor and/or metastatic sites, so they can represent the whole tumor, whereas a micro-biopsy tissue cannot, and this is particularly important for the great intra-tumoral heterogeneity of NB [15]. This aspect underlines the relevance of analyzing exo-DNA for an eventual personalized medicine. In exo-DNA samples at relapse, we observed the majority of mutations detected at diagnosis, plus an increased number of somatic SNVs such as mutations of *ALK*, *TP53*, and *RAS*/*MAPK* genes. Perhaps the specific SNVs observed at the time of relapse were present as a minor subclone at diagnosis and were not detected by standard coverage WES. The exo-DNA at the time of relapse has somatic SNVs with a higher frequency than the exo-DNA at onset of disease, and probably this is an indicator of clonal evolution. Some genes involved in the RAS/MAPK pathway have somatic SNVs in exo-DNA at the time of relapse, allowing the identification of mechanisms involved in NB progression and acquired resistance [13]. The study of SNVs detected in exo-DNA at the time of relapse, and the chance to identify these mechanisms as soon as possible, could be particularly important for the therapeutic treatment of NB patients. Interestingly, the TML values of exo-DNA both at onset and at relapse are higher than values of tumor DNA. TML has been shown to be a predictor for therapy response [28]. Koeppel and co-authors explained how the advantage of WES over targeted resequencing is not primarily the identification of new variants, but the possibility to obtain a global view of mutations at the target level too. In addition, it seems that our NB patients with high TML values in exo-DNA had a worse outcome than those with lower values. Furthermore, the ability to obtain biomarkers such as TML using non-invasive samples increases the interest of exo-DNA analysis. We observed in exo-DNA at onset hotspot mutations of genes not typically associated with NB such as *TYW1* and *DPP6*. The *TYW1* gene encodes for an iron-sulfur protein with protective functions against the adverse iron effects on neuronal functions [34,35]. The *DPP6* gene encodes for the dipeptidyl aminopeptidase-like protein 6, promoting cell surface expression of the potassium channel, and generating a current gradient critical for the regulation of dendritic excitability in hippocampal neurons [36]. We observed mutations also in *THSD7A* gene, coding for a new potential tumor antigen that might represent a putative therapeutic target for cancer therapy [37]. The *OR8G5* gene encodes for the olfactory receptor 8G5, a G-protein-coupled receptor mainly expressed in olfactory sensory neurons [38]. Moreover, olfactory receptors may be expressed also in non-olfactory tissues, likely representing new tumor markers [39]. Although these mutations are not currently associated with NB, they could be evaluated further.

Our study has several limitations, such as the relatively small number of exo-DNA samples and the not availability of the corresponding primary tumor DNA for all patients. Nevertheless, here we provide new insights into liquid biopsy subject, to try to achieve a finest personalized medicine for children affected by NB. Future works based on a larger NB samples size are required to confirm present results.

## 4. Materials and Methods

### 4.1. Patients and Samples

Nineteen patients with a diagnosis of NB were collected at BIT-NB Biobank of IRCCS Gaslini, Genova, Italy. Of these, fourteen were high-risk, and five were intermediate or low risk as defined by the International Neuroblastoma Risk Group (INRG) classification system [5]. Inclusion condition for the patients analyzed in this study was the availability of plasma obtained at onset of the disease. For four patient’s, plasma samples collected at the time of relapse were also available. The clinical data were derived from the Italian Neuroblastoma Registry, where the clinical characteristics of NB patients were saved in a pseudo-anonymized manner. Written informed consent was obtained from children’s parents to report each case. This study was undertaken in accordance with the ethical principles of the Declaration of Helsinki, and it was approved by the Italian Institutional Ethics Committee (Measure n° 270/17 related to the clinical study protocol IGG-NCA-AP-2016).

### 4.2. Sample Collection and Processing

NB samples content was confirmed by review of hematoxylin and eosin-stained tumor sections by the local pathologists. Tumor DNAs were extracted from fresh NB tissue using MasterPure DNA Purification Kit (Lucigen, Middleton, WI, USA), according to manufacturer’s instructions. Genomic germline DNA was extracted for each NB patient from peripheral blood leukocytes using the QIAamp DNA Blood Midi Kit (QIAGEN, Hilden, Germany). Blood samples were collected in EDTA tubes and plasma was separated from blood by centrifugation at 1600× *g* for 10 min at 4 °C twice, followed by careful aliquoting and freezing at −80 °C within 1 to 24 h [40].

### 4.3. Exo-DNA Purification and Quantification

We isolated exosomes from nineteen plasma NB samples collected at onset of disease and, for four patients, at the time of relapse too, as previously described [19]. Exosomes were isolated with the specific Exo-RNeasy serum/plasma Midi Kit (QIAGEN). The exosomes were incubated in 200 µL PBS with 1 µL DNase I (1 unit/µL) (Promega, Madison, WI, USA) at 37 °C for 30 min. Then, 5 µL of DNase stop solution (Promega) was added, and the exosomes were heated at 65 °C for 5 min. Following, the exosomes were washed and resuspended in 200 µL PBS [41]. Exosomes were lysed with Triton X-100 (2%), SDS (0.1%) and Buffer AL (QIAGEN) as described by Vagner et al. [42]. Finally, the exo-DNA was extracted using the QIAamp MinElute ccfDNA Mini Kit (QIAGEN). Exo-DNA quantity and quality were assessed with 1× dsDNA HS assay on Qubit 4.0 fluorometer (ThermoFisher, Waltham, MA, USA) and with high sensitivity DNA assay on Agilent Bioanalyzer (Agilent Technologies, Santa Clara, CA, USA), respectively.

### 4.4. Genomic Profiling of NB Primary Tumors

DNA from fifteen NB primary tumors was tested by high-resolution oligonucleotide a-CGH using the 4 × 180 K Kit (Agilent Technologies) with a mean resolution of approximately 25 kb [43]. The altered chromosomal regions were detected using the algorithm ADM-1 (threshold 10) with 0.5 Mb window size to reduce false positives. Chromosome positions were determined using GRCh38/hg19 (UCSC Genome Browser, http://genome.ucsc.edu, release 7 July 2000), accessed on 5 January 2021.

### 4.5. Library Construction and Exome Capture of Exo-DNA

Quality control of gDNA was performed using the DNA Broad Range Qubit Assay (ThermoFisher) and the 4150 TapeStation System (Agilent Technologies). Samples were sheared to 300 bp using the Covaris S220 instrument (Covaris, Woburn, MA, USA) according to the manufacturer’s protocol. Library construction was performed using the KAPA Hyper Prep (Roche, Basel, Switzerland) with 50 ng or all the available amount of sheared DNA as input for each sample. Both exo-DNA at onset and exo-DNA at relapse were adapter ligated with xGen Dual Index UMI Adapters (IDT, Coralville, IA, USA). Library quality control was performed using the DNA Broad Range Qubit Assay (ThermoFisher) and the 4150 TapeStation System (Agilent Technologies). Exomes were captured using the Twist Human Core Exome + RefSeq (Twist Bioscience, San Francisco, CA, USA) following the manufacture’s recommendation. Enriched libraries were quantified by real-time PCR using the KAPA Library Quantification Kit for Illumina platforms on the QuantStudio3 Real-Time PCR Systems (ThermoFisher) and polled at equimolar concentrations. Sequencing was done on the Novaseq 6000 platform (Illumina, San Diego, CA, USA) using the 150 bp paired-end mode.

### 4.6. Bioinformatics Pipeline

FastqQ files were quality controlled using FastQC (http://www.bioinformatics.babraham.ac.uk/projects/fastqc/, accessed on 30 October 2020). Low quality nucleotides and adaptors have been trimmed using sickle vl.33 (https://github.com/najoshi/sickle, accessed on 30 October 2020) and scythe v0.991 (https://github.com/vsbuffalo/scythe, accessed on 3 November 2020), respectively. Reads were consequently aligned to the reference human genome sequence version GRCh38/hg38 using BWA-MEM v0.7.15 (https://arxiv.org/abs/1303.3997, accessed on 3 November 2020). For the exosome and tumor samples processed with UMI, fgbio v1.1.0 was used to first integrate the UMI information into the BAM files and to group the reads by UMI using the “Annotate Bam with Umis” and “Group Reads by Umi” functions, respectively. Consensus reads based on UMI were then generated through the “Call Molecular Consensus Reads” function (using parameters: —error-rate-post-umi = 30 and —mim-reads = 1) The consensus reads were then re-mapped to the human references genome. All generated BAM files were cleaned by local realignment around insertion–deletion sites, duplicate marking (only for the samples processed without UMI), and recalibration using Genome Analysis Toolkit v3.8.1.6. Overlapping regions of the BAM file were clipped using BamUtil v1.4.14 to avoid counting multiple reads representing the same fragment. CallableLoci in GATK v3.8 was used to identify callable regions of the target (genotypability), with minimum read depths of 3 and 10. CollectHsMetrics by Picard v2.17.10 was used to calculate fold enrichment and FOLD 80 penalty values to determine enrichment quality. Somatic variant calling was then performed with Mutect2 (GATK v4.1.9.0) in the “tumor with matched normal” mode, considering as “tumor” the exo-DNA and tumor samples, and as “normal” the germline genomic DNA (gDNA). As a preliminary step, a “panel of normal” VCF file was created using all the germinal gDNA samples to further refine the somatic analysis. Variants were then filtered by quality (filter PASS) and target design region. Annotation and prioritization of variants was carried out using VarSeq software (GoldenHelix, Bozeman, MT, USA). The annotation process included databases of somatic mutations (COSMIC v90, TCGA Variants 3 March 2019, GHI, ICGC Simple Somatic Mutations 28, GHI, CIViC—Variant Clinical Evidence Summaries 1 July 2020, WUSTL, CIViC—Genes 1 July 2020, WUSTL), the RefSeq Genes 109 Interim v2.1 NCBI and the dbSNP 153 NCBI databases. A flag was applied for variants present in more than one tissue in the same individual.

#### 4.6.1. Copy Number Variations (CNVs)

CNVs in exo-DNA and in tumor samples were identified using the tool EXCAVATOR2 with default setting, pairing each tumor DNA and exo-DNA with the corresponding gDNA sample. Then, results were filtered using a selected list of genes known to be recurrently mutated in NB (*ALK*, *TERT*, *ATRX*, *LIN28B*, *TP53*, *SMARCA4*, *CDKN1B*, *PHOX2B*, *CHD5*, *MYCN*, *SHANK2*, *PTPRD*, *FGFR1*, *PTPN11*, *NF1*, *NRAS*, *KRAS*, and *BRAF*) [9].

#### 4.6.2. Tumor Mutation Load (TML)

Tumor mutation load (TML) of exo-DNA and of tumor DNA samples was calculated extracting the number of all the non-synonymous SNVs from these samples and dividing it by the target design length (Mb).

#### 4.6.3. Enrichment Analysis

An enrichment analysis was performed in order to identify the most mutated genes in the exo-DNA and in the DNA from tumor samples among all patients. The annotated VCF files were processed by the MutEnricher v1.3.1 software (https://rdcu.be/b51ka, accessed on 19 November 2020) using the “coding” parameter to generate *p*-values of enrichment for both hotspot sites and genic regions.

#### 4.6.4. Concordance Rate

Common CNVs shared between the exo-DNA and tumor DNA were retrieved using the SnpSift v4.3 tool. A concordance rate (percentage of concordance) was calculated considering the mean number of common CNVs identified for each pair exo-DNA and tumor DNA among all individuals.

## 5. Conclusions

Here we give evidence, for the first time, that the double stranded DNA contained in exosomes derived from plasma samples of NB patients represents the whole tumor DNA. Exo-DNA can be exploited to identify SNVs and CNVs present in parental NB cells. The possibility to isolate and enrich NB-derived exosomes from plasma using surface markers, and to extract exo-DNA quickly and easily, give this method a significant translational potential in the clinic. Exo-DNA can be an attractive circulating biomarker for NB molecular diagnostic and for clinical outcome prediction. Indeed, exo-DNA can be useful to identify early mechanisms of acquired resistance, such as mutations of *ALK*, *TP53*, and *RAS*/*MAPK* genes, occurring in relapsed patients. Future clinical studies to define exo-DNA utility in personalized NB therapy are warranted.

## Figures and Tables

**Table 1:** Patients’ characteristics and primary tumor DNA genomic profile analysis.

Case	Risk Group	Age at Diagnosis (months)	Relapse	*MYCN* Status	Genomic Profile by a-CGH	Chromosome Alterations	Last Follow-Up
**1**	M	61	Yes	Gain	Segmental	2p+, +4, +6, +7, +8, +13, +18, 17q+, 11q−, 1q+, 11q+, 12q+, 14q+, +20, 21q−, Xp+, −Y	Alive with evidence of disease
**2**	L2	34	No	Single copy	Numerical	−3, +6, −9, −11, +13, −14, −16, +17, +18, −19, −22, −X	Alive with evidence of disease
**3**	L2	165	No	Single copy	Segmental	7p−, 19p−, 19q−, 21q−	Alive
**4**	L2	1	No	Single copy	Numerical	−3, −4, +7, −9, −10, −11, +14, −16, +17, +18, −X, −Y	Alive
**5**	M	118	No	Single copy	Segmental	4q+, 10p+, 17p−, 17q+, 19p+, 22q−	Alive with evidence of disease
**6**	L2	28	No	Single copy	Numerical	+6, + 7, +13, +15, +17	Alive
**7**	M	45	No	Amplified	Segmental	1p−, 2p+, 4p−, 12q+, 17q+, 19p−	Dead
**8**	M	17	Yes	Single copy	Segmental	4p−, +6, +7, +13, −9, −10, −11, −12, −14, +15, 16p−, 16q−, +17, −X, +Y	Dead
**9**	M	88	Yes	Amplified	Segmental	2q−, 7p+, 7q+, 11q−, 11q+, 12q+, 17q+, 19q−, Xp+	Dead
**10**	M	64	No	Amplified	Segmental	+1, 2p+, +3, +7, −5, −8, 17q+, 11q−	Dead
**11**	M	45	Yes	Single copy	Not performed	-	Dead
**12**	M	70	No	Amplified	Not performed	-	Dead
**13**	M	58	No	Gain	Segmental	1p+, 2p+, 3p−, 5q+, 7q+, 13q+,14q−, 17q+, 11q−, 18p−, 19q−, −Y	Alive with evidence of disease
**14**	M	134	No	Single copy	Segmental	1q+, +5, +7, +12, +13, +17, 17q+, +18	Alive with evidence of disease
**15**	M	24	No	Amplified	Not performed	-	Dead
**16**	M	212	No	Single copy	Segmental	−1, 2p+, −3, 9p−, +10, +11, +12, 14q−	Alive with evidence of disease
**17**	M	82	No	Single copy	Not performed	-	Alive
**18**	M	46	No	Gain	Segmental	2p+, 2q+, 3p−, 4p−, +7, 7q+, −8, 8p−, 9p+, +12, 17q+, 11q−, +18, 19p−, 22q+, −X, Xq−	Alive with evidence of disease
**19**	Ms	10	Yes	Single copy	Segmental	1p−, −14, 18p−, 19q−, −Y	Alive with evidence of disease (progression and relapse)

**Table 2:** Genotypability values of whole-exome sequencing (WES) managed on genomic blood DNA (gDNA), tumor DNA, and exosomal DNA (exo-DNA).

Chromosome	Genomic DNA	Tumor DNA	Exo-DNA at Onset	Exo-DNAat Relapse
	%PASS	%PASS	%PASS	%PASS
**Chr1**	83.30	87.42	89.21	82.26
**Chr2**	84.21	88.53	90.26	82.35
**Chr3**	89.54	90.65	90.51	87.66
**Chr4**	91.15	92.44	93.57	90.54
**Chr5**	79.09	81.07	80.79	78.28
**Chr6**	93.58	94.86	95.61	93.53
**Chr7**	85.87	88.75	89.79	84.72
**Chr8**	78.87	79.86	81.1	77.98
**Chr9**	83.75	85.78	86.69	82.88
**Chr10**	89.93	93.23	95.11	89.18
**Chr11**	91.73	93.98	96.04	91.00
**Chr12**	92.21	94.27	96.4	91.40
**Chr13**	90.75	92.76	97.75	90.81
**Chr14**	94.87	97.14	97.61	93.77
**Chr15**	78.69	82.83	83.75	77.87
**Chr16**	77.94	81.04	82.63	76.47
**Chr17**	91.57	93.52	94.11	90.38
**Chr18**	88.03	91.49	96.52	88.92
**Chr19**	97.02	97.21	97.2	96.36
**Chr20**	98.48	98.42	98.26	97.91
**Chr21**	71.48	72.96	73.41	70.49
**Chr22**	87.22	89.93	92.67	87.08
**ChrX**	83.30	88.83	89.27	88.11

**Table 3:** Somatic SNVs identified in tumor DNA and in exo-DNA samples.

N° Cases	1	2	3	4	5	6	7	8	9	10	11	12	13	14	15	16	17	18	19
Genes of interest																			
*ALK* c.3824 G > A	X								X				X						
*ALK* c.3522 C > A						X					X		X						
*ALK* c.4587 C > G																			
*ALK* c.4338 G > T																			
*ALK* c.3509 T > A								X											
*ALK* c.1260 T > C																			
*ALK* c.1853 G > A																			
*ALK* c.1827 G > A																			
*CHD5* c.4789 G > A																			
*CHD5* c.1857 C > T																			
*CHD5* c.635 C > T																			
*CDKN1B* c.326 T > G																			
*PHOX2B* c.752 C > T																			
*PHOX2B* c.722_738 del																			
*DENND3* c.2053 G > T											X								
*TP53* c.811 G > A																			
*ATRX* c.3028 G > T																			
*TERT* c.510 G > T																			
*TERT* c.2297_2299 del																			
*BRAF* c.1803 A > T																			
*BRAF* c.1741 A > T																			
*MYCN* c.131 C > T								X											
*SHANK2* c.2900 A > G																			
*SHANK2* c.1175-5810																			
*SHANK2* c.5404 G > A																			
*SHANK2* c.122 C > A																			
*PTPRD* c.1335 T > C																			
*FGFR1* c.1625 del A																			
*FGFR1* c.1638 C > G												X							
*PTPN11* c.1508G > T																			
*KIT* c.2394 C > T								X											
*PLAG1* c.1372 C > A								X											
*KCNJ12* c.167 A > C								X											

Legend: 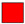 Mutation detected only in tumor DNA; 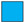 Mutation detected only in exo-DNA at onset; 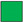 Mutation detected only in exo-DNA at relapse; 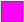 Concordant mutation in tumor DNA and in exo-DNA at onset; 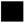 Concordant mutation in tumor DNA and in exo-DNA at relapse; **X**: SNVs frequently associated with NB.

**Table 4:** The 20 most frequently mutated genes in exo-DNA and in tumor DNA.

Exo-DNA at Onset		Exo-DNA at Relapse		Tumor DNA	
Gene	FDR_BH *p*-Value	Gene	FDR_BH *p*-Value	Gene	FDR_BH *p*-Value
*TYW1*	9.79 × 10^−^^29^	*AHNAK2*	0.00075	*ALK*	4.18 × 10^−8^
*ALK*	3.02 × 10^−^^9^	*OR52R1*	0.00075	*PRDM2*	4.7 × 10^−5^
*MUC16*	7.44 × 10^−^^9^	*OR10H3*	0.00075	*NRXN3*	4.7 × 10^−5^
*DPP6*	9.93 × 10^−^^9^	*C17orf78*	0.00075	*SI*	4.7 × 10^−5^
*OR8G5*	3.26 × 10^−^^8^	*DEGS1*	0.00075	*OTOF*	0.000119
*ALPK1*	5.92 × 10^−^^6^	*C5orf38*	0.00075	*MXI1*	0.00134
*MYH4*	6.75 × 10^−^^6^	*THEGL*	0.00114	*C5orf47*	0.00134
*LOC105370980*	7.87 × 10^−^^6^	*CPO*	0.00114	*TMEM198*	0.00134
*NCKAP5L*	9.28 × 10^−^^6^	*CYP3A43*	0.00114	*ASCL2*	0.00134
*FLG*	1.99 × 10^−^^5^	*RETSAT*	0.00114	*C1QTNF7*	0.00134
*TRIOBP*	1.99 × 10^−^^5^	*C16orf89*	0.00114	*AP1M1*	0.00134
*NPIPB2*	1.99 × 10^−^^5^	*GTPBP4*	0.00114	*ENO3*	0.00134
*DTD2*	1.99 × 10^−^^5^	*TYR*	0.00114	*CLEC9A*	0.00134
*PSG9*	2.35 × 10^−^^5^	*SLC2A10*	0.00114	*EED*	0.00134
*FRAS1*	3.18 × 10^−^^5^	*C14orf39*	0.00165	*SRL*	0.00134
*HYDIN*	3.55 × 10^−^^5^	*TAP1*	0.00185	*NPTXR*	0.00134
*MUC4*	3.55 × 10^−^^5^	*NUAK1*	0.00185	*P2RY2*	0.00134
*TMEM14B*	4.46 × 10^−^^5^	*DNAI1*	0.00197	*LCE3D*	0.00134
*ASB11*	4.55 × 10^−^^5^	*DTNA*	0.00205	*LCE2D*	0.00134
*TAF11*	4.55 × 10^−^^5^	*NPAS4*	0.00222	*MYCN*	0.00134

**Table 5:** Hotspot regions detected in exo-DNA at onset.

Exo-DNA at Onset	FDR_BH *p*-Value
chr6:41198279-41198279 (*TREML2* gene)	3.35 × 10^−17^
chr7:154053011-154053011 (*DPP6* gene)	3.33 × 10^−^^18^
chr7:154052929-154052929 (*DPP6* gene)	3.87 × 10^−18^
chr7:11831836-11831843 (*THSD7A* gene)	1.94 × 10^−10^
chr7:67067402-67067402 (*TYW1* gene)	9.61 × 10^−^^59^
chr11:124265668-124265676 (*OR8G5* gene)	2.85 × 10^−16^
chr12:123937252-123937252 (*CCDC92* gene)	1.2 × 10^−17^
chr14:31457378-31457378 (*DTD2* gene)	1.2 × 10^−17^
chr19:42740350-42740352 (*PSG3* gene)	1.14 × 10^−10^

**Table 6:** Genes displaying copy number variations (CNVs) in exo-DNA and in tumor DNA.

Gene.	Exo-DNA at Onset	Exo-DNA at Relapse	Tumor DNA
*NF1*	7	3	2
*BRAF*	5	1	3
*MYCN*	6	1	2
*ALK*	5	1	2
*SHANK2*	6	0	2
*PTPN11*	4	0	3
*TP53*	3	0	4
*ATRX*	1	1	4
*CDKN1B*	4	0	2
*KRAS*	3	0	2
*TERT*	3	1	1
*CHD5*	2	1	0
*PHOX2B*	1	0	2
*PTPRD*	1	0	1
*SMARCA4*	1	0	1
*FGFR1*	1	0	1

## Data Availability

All the data are reported in the present paper and in the supplementary materials.

## Supplementary Materials

The following are available online at https://www.mdpi.com/article/10.3390/ijms22073667/s1.

## Abbreviations

NB	Neuroblastoma
*MYCN*	v-myc myelocytomatosis viral related oncogene, neuroblastoma derived
*ALK*	Anaplastic Limphoma Kinase
RAS	Rat Sarcoma
INRG	International Neuroblastoma Risk Group
a-CGH	Array-Comparative Genome Hybridization
chr	Chromosome
mAb	Monoclonal antibody
dsDNA	Double stranded DNA
gDNA	Genomic DNA
cfDNA	Cell-free DNA
ctDNA	Circulating tumor DNA
exo-DNA	Exosomal DNA
WES	Whole Exome Sequencing
UMI	Unique Molecular Identifier
SNV	Single Nucleotide Variant
CNV	Copy Number Variation
VAF	Variant Allele Frequency
TML	Tumor Mutation Load
VCF	Variant Call Format
COSMIC	Catalogue of Somatic Mutations in Cancer
