# Supplementary material for: Exosomes from Plasma of Neuroblastoma Patients Contain Doublestranded DNA Reflecting the Mutational Status of Parental Tumor Cells
Source: Int J Mol Sci. 2021 Apr 1;22(7):3667. doi: 10.3390/ijms22073667 (PMC8036333; doi:10.3390/ijms22073667)
Supplement: Supplementary file 1 [file ijms-22-03667-s001.zip › Supplementary Material.docx]

Supplementary Material, Degli Esposti C. et al.

**
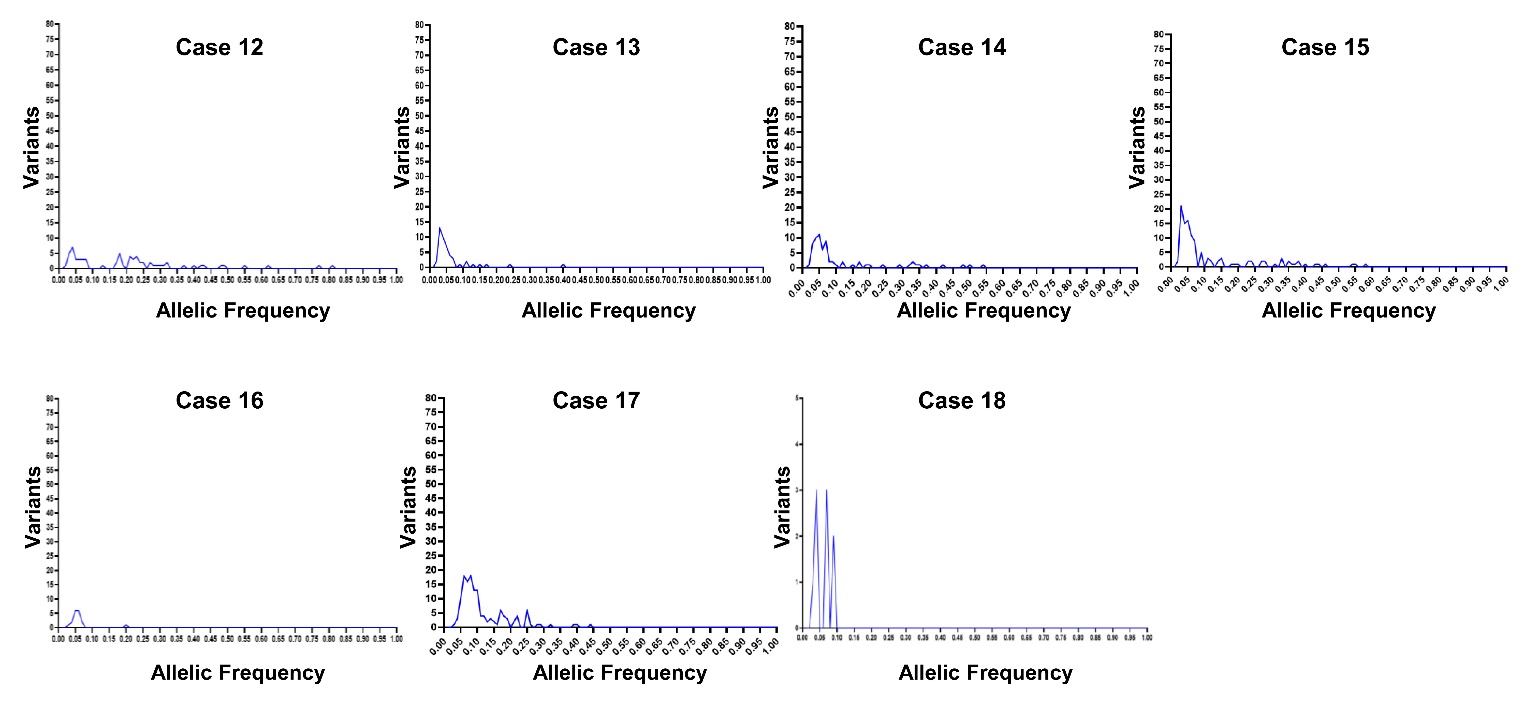
**

Supplementary Figure S1.

Somatic SNVs frequency in NB cases with exo-DNA at onset only.
